# Aortic Dissection and Cardiac Tamponade in an Elderly Female With Cushing’s Syndrome: A Fatal Case

**DOI:** 10.7759/cureus.83744

**Published:** 2025-05-08

**Authors:** Paige O Daly, Sat Paul Singh, Chelsea Bengson, Camelia Pana

**Affiliations:** 1 Research, Edward Via College of Osteopathic Medicine, Blacksburg, USA; 2 Internal Medicine, Riverside Health System, Newport News, USA

**Keywords:** aortic dissection, autopsy, cardiac tamponade, cardiology, cardiovascular emergency, cushing’s syndrome, endocrinology, hypercortisolemia, pericardial effusion, sudden death

## Abstract

A 77-year-old female with multiple cardiovascular comorbidities presented with increased work of breathing and was found to have hypotension, bradycardia, and hypoxia. Despite initial stabilization, the patient's condition declined, leading to a sudden cardiac arrest. Postmortem examination revealed the cause of death as aortic dissection, with exsanguination into the pericardium and left pleural space. This case illustrates the value of considering aortic dissection in patients with a history of Cushing's syndrome, even when their presenting symptoms are nonspecific.

## Introduction

Aortic dissection is a life-threatening cardiovascular emergency characterized by a tear in the inner layer of the aorta, allowing blood to flow between the layers of the aortic wall [1]. This condition is associated with significant morbidity and mortality, particularly when diagnosis and treatment are delayed.

The incidence of aortic dissection is estimated to be between three and six cases per 100,000 person-years. However, some studies, such as one in Japan that employed systematic post-mortem imaging, report higher incidences at 17.6 cases per 100,000 person-years [2-3]. Aortic dissection is more prevalent in men, with a male-to-female ratio of approximately 1.5:1 to 3:1, and typically affects individuals between 50 and 70 years of age [4-5].

Aortic dissections are classified into type A, the ascending aorta, and type B, the descending aorta. Type A dissections are more common, accounting for 60-67% of cases [4,6].

The classic presentation of aortic dissection includes sudden, severe chest or back pain, often described as tearing or ripping in nature. In addition to the more commonly recognized symptoms of aortic dissection, such as severe chest pain, hypotension or syncope, neurological symptoms, and pulse deficits, dysphagia can also be a symptom of aortic dissection [7]. However, atypical presentations can make diagnosis challenging, especially in patients with multiple comorbidities.

Established risk factors for aortic dissection include hypertension, advanced age, male sex, smoking, and connective tissue disorders like Marfan syndrome. Less commonly recognized risk factors include a history of cocaine use, pregnancy, and certain inflammatory conditions [4,8]. Recent evidence suggests a link between endocrine disorders, particularly Cushing's syndrome, and an increased risk of aortic dissection [9].

The diagnosis of aortic dissection remains challenging, particularly in patients presenting with atypical symptoms or those with multiple comorbidities that may mask or mimic the signs of aortic pathology. Imaging modalities such as CT angiography (CTA), magnetic resonance angiography (MRA), and transesophageal echocardiography (TEE) play crucial roles in confirming the diagnosis. However, the initial suspicion often relies on clinical acumen and recognition of risk factors.

This case report presents an unusual and complex case of aortic dissection in a 77-year-old female with a history of Cushing's syndrome and multiple other cardiovascular comorbidities. The patient's atypical presentation, characterized by respiratory distress and hemodynamic instability, posed significant diagnostic challenges. By examining this case in detail, we aim to highlight the importance of considering aortic dissection in patients with a history of Cushing's syndrome, even when presenting with nonspecific symptoms, and to discuss the potential long-term cardiovascular risks associated with this endocrine disorder.

## Case presentation

A 77-year-old female presented to the emergency department with increased work of breathing. Her past medical history was significant for coronary artery disease, dyslipidemia, hypertension, paroxysmal atrial fibrillation (on Eliquis), hyperlipidemia, Cushing's syndrome status post adrenalectomy, heart failure with preserved ejection fraction (55-60%), and nonrheumatic aortic valve insufficiency. Her home medications included diltiazem (Cardizem) 240 mg once daily and propranolol (Inderal) 60 mg once daily.

The patient reported dysphagia for several days prior to presentation and had experienced a restless night. Her home health aide noticed increased work of breathing, prompting the call to emergency medical services. On arrival, the patient required five 5 L of oxygen via nasal cannula. She had no recent upper respiratory infection symptoms or sick contacts. Initial examination revealed a Glasgow Coma Scale of 15, with the patient appearing out of breath. Her blood pressure was notably low, with a mean arterial pressure of 49 mmHg. Her other vital signs were remarkable for bradycardia with a heart rate as low as 30 beats per minute and hypoxia with SpO2 as low as 89%. Due to the bradycardia, 500 mL of normal saline was administered along with 1 mg of atropine. The patient was placed on bilevel positive airway pressure for respiratory support. These interventions improved oxygen saturation, blood pressure, and heart rate, with the latter increasing to the 70-90s range.

Physical examination revealed an ill-appearing, diaphoretic patient in acute distress with a large body habitus. She exhibited respiratory distress with diminished breath sounds bilaterally, increased work of breathing, and tachypnea. The cardiovascular exam was notable for an irregular rhythm and bradycardia, consistent with atrial fibrillation. Edema was present, consistent with heart failure. The patient was only oriented to self. There were no other pertinent findings on the detailed physical exam.

Laboratory values upon admission are located in Table 1. Laboratory studies showed an elevated brain natriuretic peptide (BNP) of 613, significantly increased from 131 two months prior, suggesting acute cardiac stress. Troponin was elevated at 30. A point-of-care venous blood gas was reassuring, with pH within normal limits and no excess pCO₂.. Complete blood count showed mildly elevated white blood cells (WBC) at 11.1k/mcL, without anemia. Hemoglobin of 12.8 gm/dL and platelet count of 162 k/mcL were within normal limits. Other notable lab findings included a comprehensive metabolic panel (CMP) revealing hyponatremia (127 mmol/L), elevated blood urea nitrogen (BUN) (33 mg/dL), and creatinine of 1.2 mg/dL, consistent with the patient's baseline. All other lab values in the CMP were within normal limits. Thyroid-stimulating hormone and free T4 were within normal limits, too, and the influenza and COVID-19 tests were negative.

**Table 1:** Patient's admission laboratory values WBC: white blood cell, RBC: red blood cell, BUN: blood urea nitrogen, eGFR: estimated glomerular filtration rate, pH: potential of hydrogen, PCO2: partial pressure of carbon dioxide, PO2: partial pressure of oxygen, SO2: sulfur dioxide

Parameters	Patient's values	Reference range	Units
WBC	11.1	4.5-10	k/mcL
RBC	4.19	4.7-6.1	million cells/mcL
Hemoglobin	12.8	12.1-15.1	g/dL
Hematocrit	40	36-48	%
Mean cell volume	95.3	80-100	fL
Mean cell hemoglobin	30.5	27-32	pg
Red cell distribution width	15.1	11.5-14.5	%
Platelet count	162	150-400	k/mcL
Neutrophil automated absolute number	8.8	1.5-7.7	k/mcL
Sodium level	127	135-145	mmol/L
Potassium level	4.8	3.5-5.0	mmol/L
Chloride	95	96-106	mmol/L
Carbon dioxide	23	23-29	mmol/L
Anion gap	9	4.0-12.0	mEq/L
BUN	33	6.0-20.0	mg/dL
Creatinine	1.2	0.6-1.1	mg/dL
Magnesium level	2	1.6-2.6	mg/dL
eGlomerular filtration rate	52	>90	mL/min/1.73 m^2
Troponin I, high sensitivity	30	0-0.04	ng/mL
B-type natriuretic peptide	613	<100	pg/mL
Bilirubin total	1.9	0.3-1.2	mg/dL
Bilirubin direct	0.9	0.1-0.3	mg/dL
pH venous	7.359	7.32-7.43	-
PCO2 venous	41.5	35-45	mmHg
PO2 venous	60	25-40	mmHg
SO2 venous	90	70-80	%
Bicarbonate venous	23.4	23-29	mm
Thyroid-stimulating hormone	2.252	0.4-4.0	mIU/L
Free T4	1.48	0.8-1.9	ng/dL

**Figure 1:**
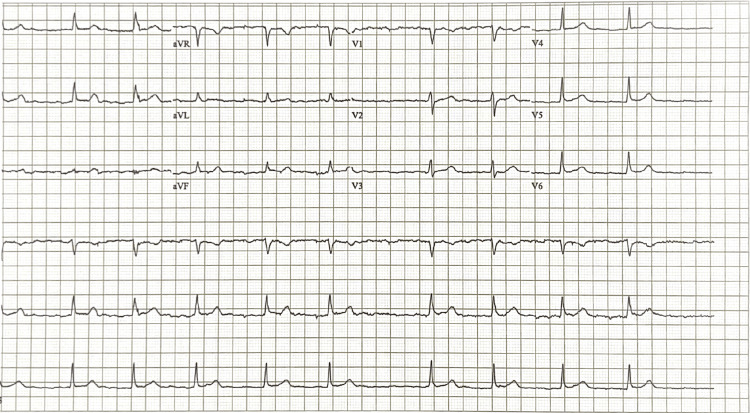
EKG showing an irregular, bradycardic rhythm with a ventricular rate of 52 bpm and a QTc of 414 ms and minimal ST elevation in leads I and II without reciprocal depression EKG: electrocardiogram

**Figure 2:**
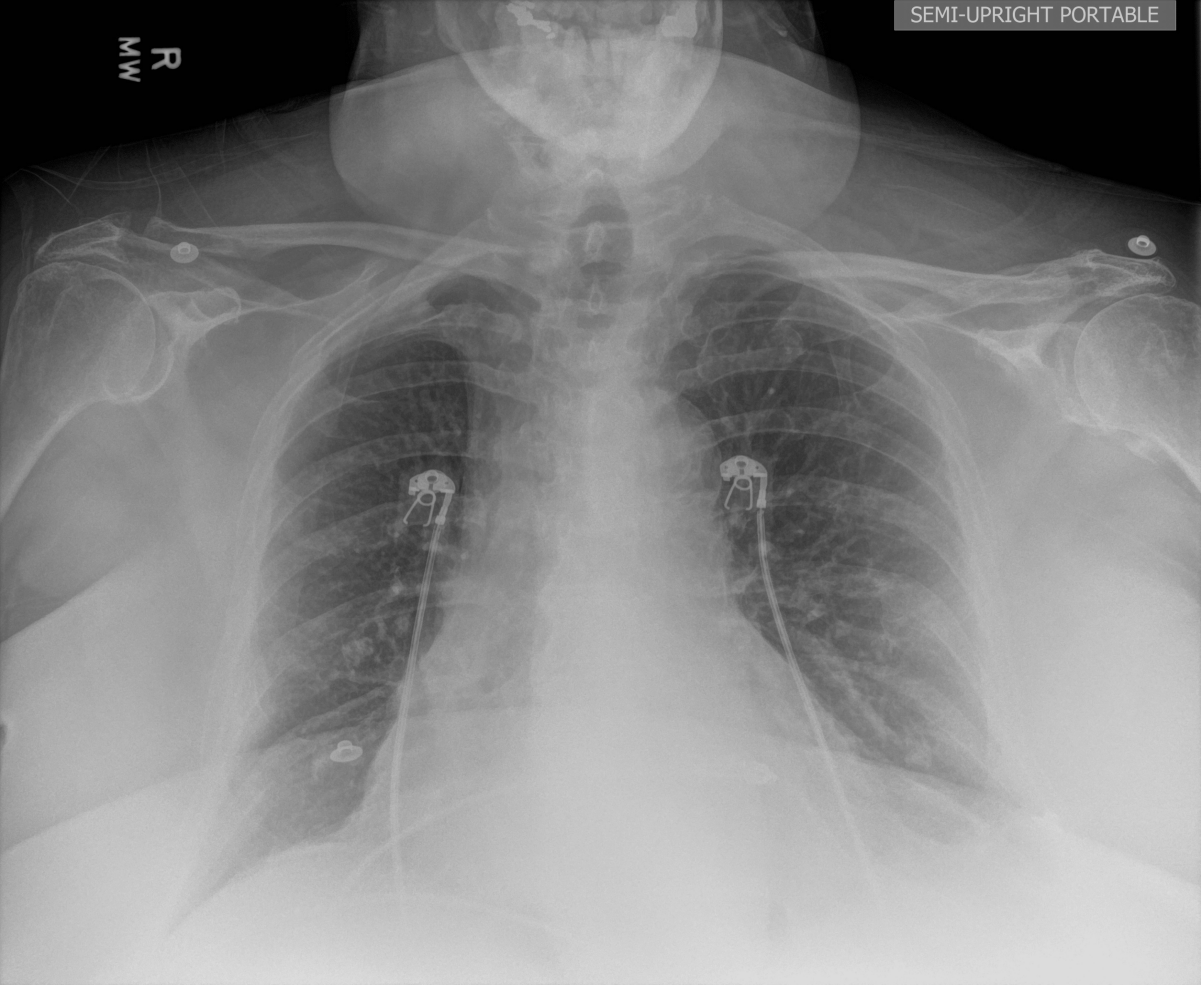
Upright chest X-ray showing a large cardiac silhouette consistent with heart failure, mild vascular congestion, left basilar atelectatic changes, and a flattened diaphragm

EKG demonstrated an irregular, bradycardic rhythm with a ventricular rate of 52 bpm and a QTc of 414 ms (Figure 1). Minimal ST elevation was noted in leads I and II without reciprocal depression. Chest X-ray revealed a large cardiac silhouette consistent with heart failure, mild vascular congestion, left basilar atelectatic changes, and a flattened diaphragm (Figure 2). CT of the head and neck without contrast showed no acute intracranial abnormalities.

Due to the patient's condition and risk of decompensation, she was admitted to the cardiac care unit for monitoring. Over the next day, the patient became somnolent but was able to be weaned off oxygen. She was found to have a urinary tract infection and was started on Zosyn. Her risperidone dose was reduced from 2 mg BID to 1 mg BID due to family concerns about potential oversedation, despite the patient not presenting with any extrapyramidal symptoms. Her hypertensive medications (Inderal and Cardizem) were stopped due to the continued low ranges of blood pressure and heart rate.

Additional pertinent labs on the second day of admission showed a downtrend of WBCs to within the normal range, an uptrend of serum sodium (but still hyponatremic at 131 mmol/L), an uptrend of BUN and creatinine to 37 mg/dL and 1.28 mg/dL, and a downtrend of troponin to 29. Cortisol was elevated at 28.7 mcg/dL.

On the third day of admission, the patient was more alert but waxing and waning at A&O (alert and oriented) x3. During this time, she had complained of persistent dysphagia and dysuria. On the physical exam, the patient was not in acute distress. Cardiovascular exam revealed a normal rate and rhythm, normal pulses, and no murmurs, rubs, or gallops. Pulmonary effort was normal with no signs of respiratory distress, and the abdomen was soft, non-tender, with normal bowel sounds. No other pertinent findings were noted during the examination. An esophagram was ordered to evaluate her swallowing difficulties.

That evening, a code blue was called for the patient. Prior to the call, the patient was alert and oriented but suddenly stopped breathing and became pulseless. Initial resuscitation efforts were started but discontinued due to the patient's DNR status, and the time of death was called.

Autopsy revealed the cause of death to be an aortic dissection with exsanguination into the pericardium and left pleural space. The autopsy report noted a ruptured pericardium with 500 mL of blood in the pericardial space and 1000 mL of serosanguinous fluid in the pleural cavity. Significant dissection was found in the ascending aorta to the arch, with intimal perforations just above the valve and an adventitial perforation 1 cm above the intimal tears. Microscopic examination showed thinning of the aortic wall with dystrophic calcifications and hemorrhage in the adventitia. The pathologist suggested that the patient's history of Cushing's disease may have contributed to the aortic dissection due to the effects of hypercortisolemia on aortic smooth muscle cells (SMCs).

## Discussion

Diagnostic challenges

This case highlights the diagnostic challenges in patients with aortic dissection and multiple cardiovascular comorbidities. The patient’s initial presentation with respiratory distress, hypotension, and bradycardia was initially attributed to her known cardiac and pulmonary conditions, leading to a delay in recognizing the underlying aortic pathology. The nonspecific nature of her symptoms further complicated the diagnostic process. The absence of classic symptoms, like sudden, severe chest or back pain, and the presence of increased work of breathing and dysphagia made the diagnosis elusive. This underscores the need for a high index of suspicion for aortic dissection in high-risk patients, even with atypical presentations.

Dysphagia, or difficulty swallowing, is a rare but clinically significant symptom associated with aortic dissection [1]. This unusual presentation arises due to extrinsic compression of the esophagus by the dilated or dissecting aorta, a condition termed "dysphagia aortica" [10]. In this patient, dysphagia was reported in the days leading up to her acute decompensation, but its potential relationship to an underlying aortic pathology was not initially recognized. This highlights the diagnostic challenge of associating dysphagia with cardiovascular emergencies, especially in patients with complex medical histories.

Aortic dissection can lead to esophageal compression as the false lumen expands or as hematoma formation occurs within the thoracic cavity [1]. This mechanical obstruction may initially manifest as difficulty swallowing solids and progress to include liquids as the compression worsens. Studies have documented that dysphagia caused by thoracic aortic aneurysm or dissection is often overlooked because it mimics more common gastrointestinal or neurologic conditions, such as gastroesophageal reflux disease (GERD) or stroke-related dysphagia [11-13]. In this case, the patient’s history of GERD and other comorbidities likely diverted attention from the possibility of an aortic etiology.

The presence of dysphagia in patients with risk factors for aortic disease should prompt consideration of imaging studies, such as CTA, which can identify thoracic aortic pathology. As demonstrated in this case, failure to recognize dysphagia as a potential symptom of aortic dissection may delay diagnosis and lead to catastrophic outcomes. Early recognition of this rare association is critical for timely intervention and improved patient outcomes.

Role of Cushing's syndrome in aortic dissection

The patient's history of Cushing's syndrome is particularly noteworthy in this case. While the patient had undergone adrenalectomy for Cushing's syndrome, the elevated cortisol level (28.7 mcg/dL) at presentation suggests ongoing hypercortisolemia. This raises important questions about the long-term cardiovascular effects of Cushing's syndrome, even after apparent treatment.

Cushing's syndrome, characterized by chronic exposure to excess glucocorticoids, has been identified as a risk factor for aortic dissection. The primary mechanism through which Cushing's syndrome increases the risk of aortic dissection is related to the effects of hypercortisolism on the SMCs and connective tissue of the aortic wall. Elevated cortisol levels can induce a phenotype switch in vascular SMCs from a contractile to a synthetic type, increasing proteolytic activity and degradation of the extracellular matrix (ECM) [14]. This weakens the structural integrity of the aortic wall, increasing susceptibility to dissection.

Additionally, hypercortisolism promotes oxidative stress and inflammation within the vascular wall. Increased levels of superoxide anions and other reactive oxygen species can damage the ECM and SMCs, compromising the aortic wall's integrity [15]. Moreover, glucocorticoids can impair the synthesis and repair of collagen and other ECM components, weakening the aortic wall and increasing the risk of dissection under hemodynamic stress [14-15].

Chronic hypercortisolemia has been shown to cause arteriosclerosis and hypertension [15]. Hypertrophic remodeling of small resistance arteries in Cushing's syndrome patients suggests a systemic effect of cortisol on vascular structure, increasing vascular stiffness and susceptibility to dissection.

Experimental models have demonstrated that cortisone can produce dissecting aortic aneurysms in animals, supporting the link between hypercortisolism and aortic pathology [14,16]. The duration of exposure to high serum cortisol levels appears more critical than the absolute cortisol levels in determining the risk of aortic dissection, highlighting the importance of the chronicity of hypercortisolism in vascular pathology [17]. These pathophysiological changes induced by hypercortisolism underline the importance of early diagnosis and treatment of Cushing's syndrome to prevent potentially life-threatening cardiovascular complications.

Implications for clinical practice

This case has several important implications for clinical practice, particularly in managing patients with a history of Cushing's syndrome and other complex comorbidities. First, clinicians must maintain a high index of suspicion for aortic dissection in these patients, even when they present with atypical symptoms such as dysphagia or respiratory distress. Recognizing subtle or uncommon signs is critical for early diagnosis. Advanced imaging modalities, such as CTA or MRA, should be considered in high-risk patients with unexplained cardiopulmonary symptoms to identify potential aortic pathology promptly. Additionally, the cardiovascular effects of Cushing's syndrome, including hypercortisolemia-induced vascular changes, may persist even after apparent treatment, necessitating ongoing surveillance and monitoring for long-term complications.

The potential for rapid clinical deterioration in cases of aortic dissection further underscores the need for vigilant monitoring of at-risk patients. This includes close observation during hospital admission and proactive management strategies to address any signs of hemodynamic instability. The case also highlights the importance of interdisciplinary care involving endocrinology, cardiology, and primary care teams to ensure comprehensive management of patients with endocrine disorders and their associated cardiovascular risks. By incorporating these considerations into clinical practice, healthcare providers can improve early detection, optimize management strategies, and potentially prevent catastrophic outcomes in vulnerable patient populations.

Future research directions

This case highlights several important areas for future research to improve the understanding and management of aortic dissection in patients with endocrine disorders such as Cushing's syndrome. Investigating the long-term cardiovascular outcomes in patients with treated Cushing's syndrome is essential to determine the extent and persistence of vascular risks, even after hypercortisolism has been addressed. Additionally, research is needed to establish optimal surveillance strategies for detecting aortic pathology in patients with a history of hypercortisolemia, including the required frequency and imaging studies. The role of biomarkers in risk stratification for aortic dissection in this population warrants exploration, as they could provide non-invasive tools to identify patients at higher risk. Finally, developing comprehensive risk prediction models incorporating endocrine disorders like Cushing's syndrome alongside traditional risk factors could enhance early identification and prevention of aortic dissection. These research directions can potentially guide clinical practice and improve outcomes for high-risk patients.

## Conclusions

This case report highlights the critical importance of maintaining a high index of suspicion for aortic dissection in patients with complex medical histories, particularly those with a history of Cushing's syndrome. The atypical presentation of increased work of breathing, without the classic symptoms of chest or back pain, underscores the need for clinicians to consider less common but potentially catastrophic diagnoses in high-risk patients. The rapid progression from initial stabilization to sudden cardiac arrest emphasizes the potentially fulminant nature of aortic dissections and the need for continuous, vigilant monitoring. The patient's history of Cushing's syndrome emerges as a crucial factor in this case, highlighting the long-term cardiovascular risks associated with hypercortisolemia, even after apparent treatment. This case serves as a powerful reminder that the effects of endocrine disorders on the cardiovascular system may persist long after the primary condition has been addressed. It calls for reconsidering long-term management strategies for patients with a history of Cushing's syndrome, potentially including more intensive cardiovascular surveillance and regular imaging studies to assess aortic integrity.

Moving forward, this case underscores the need for further research into the long-term cardiovascular outcomes of patients with treated Cushing's syndrome. It also highlights the importance of developing more effective risk stratification tools and diagnostic strategies for aortic dissection in patients with complex medical histories. By learning from cases like this, we hope to improve our ability to detect and prevent such catastrophic outcomes, ultimately enhancing patient care and saving lives in this vulnerable population.

## References

[1] Isselbacher EM, Preventza O, Hamilton Black J 3rd (2022). 2022 ACC/AHA guideline for the diagnosis and management of aortic disease: a report of the American Heart Association/American College of Cardiology joint committee on clinical practice guidelines. Circulation.

[2] Mussa FF, Horton JD, Moridzadeh R, Nicholson J, Trimarchi S, Eagle KA (2016). Acute aortic dissection and intramural hematoma: a systematic review. JAMA.

[3] Czerny M, Grabenwöger M, Berger T (2024). EACTS/STS guidelines for diagnosing and treating acute and chronic syndromes of the aortic organ. Ann Thorac Surg.

[4] Obel LM, Lindholt JS, Lasota AN (2022). Clinical characteristics, incidences, and mortality rates for type A and B aortic dissections: a nationwide Danish population-based cohort study from 1996 to 2016. Circulation.

[5] Mészáros I, Mórocz J, Szlávi J, Schmidt J, Tornóci L, Nagy L, Szép L (2000). Epidemiology and clinicopathology of aortic dissection. Chest.

[6] MacGillivray TE, Gleason TG, Patel HJ (2022). The Society of Thoracic Surgeons/American Association for Thoracic Surgery clinical practice guidelines on the management of type B aortic dissection. Ann Thorac Surg.

[7] Perheentupa U, Kinnunen I, Kujari H, Grénman R, Mäkitie AA (2010). Acute dysphagia associated with aortic dissection: a case report and review of the literature. Acta Otolaryngol.

[8] Nienaber CA, Clough RE, Sakalihasan N (2016). Aortic dissection. Nat Rev Dis Primers.

[9] Isidori AM, Graziadio C, Paragliola RM (2015). The hypertension of Cushing's syndrome: controversies in the pathophysiology and focus on cardiovascular complications. J Hypertens.

[10] Wada T, Oyama S, Ohuchi S (2021). Calcified aortic wall removal for dysphagia aortica caused by chronic traumatic aortic pseudoaneurysm. Ann Vasc Surg.

[11] Kische S, Werner D, Ince H (2012). A neglected symptom of contained aortic laceration--dysphagia aortica successfully treated by endovascular stentgrafting. Catheter Cardiovasc Interv.

[12] Wilkinson J, Euinton H, Smith L, Bull MJ, Thorpe JA (1997). Diagnostic dilemmas in dysphagia aortica. Eur Assoc Cardiothorac Surg.

[13] Liao CY, Huang SC, Wang YC, Chin HK, Tsai CC, Ben RJ, Wu HM (2015). Dysphagia aortica: a fatal delay in diagnosis. Am J Emerg Med.

[14] Zhang L, Zhou J, Jing Z, Xiao Y, Sun Y, Wu Y, Sun H (2018). Glucocorticoids regulate the vascular remodeling of aortic dissection via the p38 MAPK-HSP27 pathway mediated by soluble TNF-RII. EBioMedicine.

[15] Rizzoni D, Porteri E, De Ciuceis C (2009). Hypertrophic remodeling of subcutaneous small resistance arteries in patients with Cushing's syndrome. J Clin Endocrinol Metab.

[16] Knowlton A, Loeb E, Stoerk H, White JP, Heffernan JF (1952). Induction of arterial hypertension in normal and adrenalectomized rats given cortisone acetate. J Exp Med.

[17] Takenouchi H, Anno T, Iwamoto H (2022). Onset of aortic dissection complicated with Cushing’s disease: a case report and review of the literature. Intern Med.

